# SGLT2 Inhibitors in Long COVID Syndrome: Is There a Potential Role?

**DOI:** 10.3390/jcdd10120478

**Published:** 2023-11-29

**Authors:** Paul Zimmermann, Harald Sourij, Felix Aberer, Sian Rilstone, Janis Schierbauer, Othmar Moser

**Affiliations:** 1 Division of Exercise Physiology and Metabolism, BaySpo—Bayreuth Center of Sport Science, University of Bayreuth, 95440 Bayreuth, Germany; paul.zimmermann@arcormail.de (P.Z.); sian.rilstone@uni-bayreuth.de (S.R.); janis.schierbauer@uni-bayreuth.de (J.S.); 2 Interdisciplinary Center of Sportsmedicine Bamberg, Klinikum Bamberg, 96049 Bamberg, Germany; 3 Department of Cardiology, Klinikum Bamberg, 96049 Bamberg, Germany; 4 Interdisciplinary Metabolic Medicine Research Group, Division of Endocrinology and Diabetology, Medical University of Graz, 8036 Graz, Austria; ha.sourij@medunigraz.at (H.S.); felix.aberer@medunigraz.at (F.A.); 5 Faculty of Medicine, Department of Metabolism, Digestion and Reproduction, Imperial College London, London SW7 2AZ, UK

**Keywords:** coronavirus disease (COVID)-19, long COVID syndrome, sodium–glucose cotransporter 2 inhibitors, SGLT2 inhibitors, cytokine storm, cardiovascular disorders, long COVID and cardiovascular sequelae

## Abstract

The coronavirus disease (COVID)-19 has turned into a pandemic causing a global public health crisis. While acute COVID-19 mainly affects the respiratory system and can cause acute respiratory distress syndrome, an association with persistent inflammatory stress affecting different organ systems has been elucidated in long COVID syndrome (LCS). Increased severity and mortality rates have been reported due to cardiophysiological and metabolic systemic disorders as well as multiorgan failure in COVID-19, additionally accompanied by chronic dyspnea and fatigue in LCS. Hence, novel therapies have been tested to improve the outcomes of LCS of which one potential candidate might be sodium–glucose cotransporter 2 (SGLT2) inhibitors. The aim of this narrative review was to discuss rationales for investigating SGLT2 inhibitor therapy in people suffering from LCS. In this regard, we discuss their potential positive effects—next to the well described “cardio-renal-metabolic” conditions—with a focus on potential anti-inflammatory and beneficial systemic effects in LCS. However, potential beneficial as well as potential disadvantageous effects of SGLT2 inhibitors on the prevalence and long-term outcomes of COVID-19 will need to be established in ongoing research.

## 1. Introduction

The severe acute respiratory syndrome caused by coronavirus 2 (SARS-CoV-2) firstly described in Wuhan, China in December 2019, was spreading all over the world resulting in a global pandemic [1]. SARS-CoV-2 caused a tragic pandemic that resulted in more than 772 million infections worldwide and more than 6.9 million cumulative deaths, as registered in November 2023 [2]. Nevertheless, it is acknowledged that even in countries with developed disease notification systems, the prevalence rates of this pandemic infection have been widely underestimated [3]. Coronavirus disease 2019 (COVID-19) mainly affects the respiratory system causing acute respiratory distress syndrome; however, it is also associated with different organ system failures such as cardiac complications, i.e., infection-associated myocarditis or malignant arrhythmias, or kidney disease, as well as metabolic systemic disorders, i.e., electrolyte disturbances or multiorgan failure [1,4,5]. As the COVID-19 pandemic represents to date the greatest global health crisis, an effective, rapid, and sensitive diagnostic assessment, followed by effective treatment and management strategies, as well as preventing approaches, have been postulated to be successful for pandemic management [6]. Current management and treatment strategies are primarily based on preventive population-wide vaccination, followed by a combinational pharmacological treatment based on antiviral agents, polymerase inhibitors, proteinase inhibitors, or additional steroids and supportive oxygen therapy [6,7,8,9,10,11].

Nevertheless, long COVID syndrome (LCS)—as an often debilitating illness post an acute COVID-19 infection—is reported in at least 10% of SARS-CoV-2 infections [12]. In this context, more than 200 variable COVID-19-associated symptoms have been identified affecting multiple organ systems, mainly cardiovascular, respiratory, renal, neurological, and immunomodulatory components, including immune system dysregulation and excessive mast cell activation [12,13]. Persistent cardiopulmonary symptoms including chronic dyspnea and chest pain as well as autonomic manifestations, i.e., chronic fatigue, “brain fog”, and postural orthostatic tachycardia associated with heightened anxiety and depression are common findings in LCS altering the lives of millions of people worldwide [14,15]. A metanalysis of previous studies demonstrated a certain association between epidemiological characteristics and comorbidities and the subsequent risk of developing LCS [16]. Pre-existing comorbidities, such as chronic obstructive pulmonary disease, diabetes, ischemic heart disease, or immunosuppression have been reported as having a higher associated risk of LCS [16]. Nevertheless, up to now, a combinational treatment is estimated to be favorable for the treatment of COVID-19 [5,17], but no specific pharmacological treatment is available in LCS to relieve LCS symptoms effectively [3]. The hormone melatonin, as an activator of the nuclear factor erythroid-derived 2-linke 2 (NRF2) transcription factor, has been identified to influence positively the intracellular antioxidative status reducing oxidative stress [3]. However, this therapeutical approach requires further scientific evaluation by appropriately designed trials. Since oxidative stress in endothelial cells is the previously described first molecular pathophysiological disturbance in diabetes as well as in viral-infected cells [18], causing capillary damage, local hypoxia, and potential chronic oxidative stress, further therapeutic approaches in LCS might focus on these pathways. Next to the potential role of melatonin in LCS addressing the NRF2 pathway [19,20,21], atorvastatin with its pleiotropic effects improving endothelial function by decreasing vascular inflammation and oxidative stress has been evaluated [22].

Due to the pathophysiological molecular parallels in diabetes as well as in viral-infected cells, this narrative literature review discusses the potential role of sodium–glucose cotransporter 2 (SGLT2) inhibition therapy in the context of LCS.

The use of SGLT2 inhibitors, firstly used in people with type 2 diabetes (T2D), has been proven as an effective therapeutic approach for different types of heart failure, as defined in the universal definition of heart failure (HF) in 2021 by the European Society of Cardiology (ESC) [23]. Additionally, SGLT2 inhibitors are an established anti-hyperglycemic agent for the treatment of T2D [24,25]. Next to their role as an antidiabetic agent improving hyperglycemia in people with T2D, the SGLT2 inhibitors empagliflozin and dapagliflozin revealed a reduction in the combined risk of hospitalization or cardiovascular death in patients suffering from various types of HF with or without diabetes [26,27,28]. Additional data revealed the beneficial therapeutic effects of SGLT2 inhibitor treatment in people with chronic kidney disease (CKD) to delay disease progression and improve cardiovascular benefits [28,29], as well as additional positive effects of SGLT2 inhibitor treatment due to modest reductions in blood pressure and bodyweight [24,25,28].

Acute COVID-19 as well as LCS—in general and in the context of coexistent diseases like T2D and metabolic syndrome—represent a multiorgan and multisystem disorder [3]. Especially, LCS might be characterized by an unpredictable relapsing–remitting inflammatory response, entailing additionally activated inflammation sequelae, glucose homeostasis disturbances, hemoglobin deoxygenation, or altered immune status, and activation of the renin–angiotensin–aldosterone system (RAAS) [25,30].

Therefore, this narrative literature review discusses the rationale for investigating SGLT2 inhibitor therapy in the context of LCS.

## 2. Materials and Methods

For this narrative review, a non-systematic literature search was performed in the PubMed database between January 2023 and November 2023, searching for studies, case reports, and review articles in the context of SGLT2 inhibition therapy and LCS. Key articles from these topics of research were included based on the writing group’s decision.

## 3. The Impact of SGLT2 Inhibitors in LCS-Associated Pathophysiological Mechanisms

### 3.1. COVID-19 and LCS

**Figure 1:**
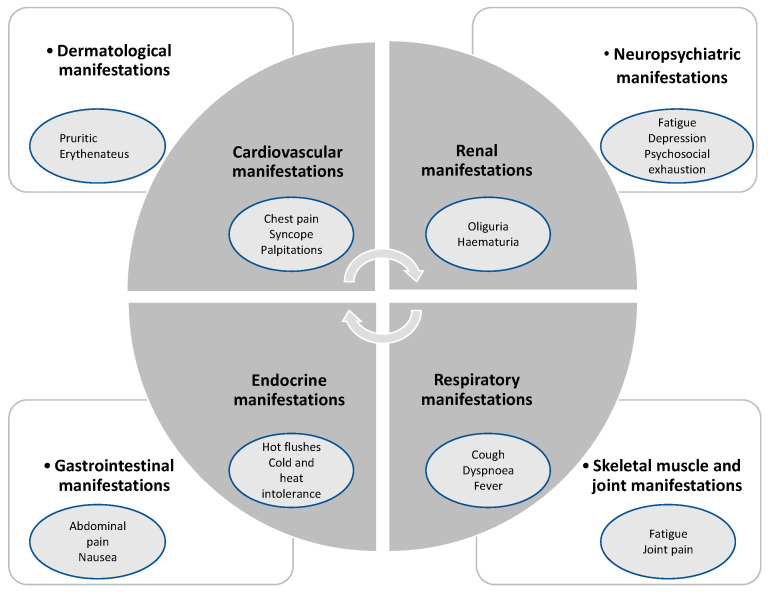
Association of diverse symptoms with LCS, inspired by Raman et al. [14].

Gaining importance in the public health system, LCS or post-acute sequelae of COVID-19 disease are characterized by persistent symptoms following SARS-CoV-2 infection [14]. The latest research in the United Kingdom (UK) estimated the number of affected patients by LCS at 1 million [31]. While there was no universally accepted definition of this pathological condition, the United Kingdom’s National Institute for Health and Care Excellence (NICE) guidelines defined LCS as the following: persistence of symptoms beyond 4 weeks after SARS-CoV-2 infection including two phases, such as ongoing symptomatic phase (4–12 weeks) and post-COVID-19 syndrome (>12 weeks) [32,33]. Additionally, the recent definition of LCS by the World Health Organization (WHO) includes a persistence of symptoms beyond 3 months after previous SARS-CoV-2 infection lasting for more than 2 months without any explanation by another illness [34]. In this context, firstly, cardiopulmonary symptoms, such as shortness of breath or intermittent chest pain presumably based on abnormalities related to myocardial inflammation, remodeling, and arrhythmias, and secondly, disturbed autonomic and neuropsychiatric manifestations, such as fatigue, headaches, brain fog, or orthostatic disorders, are previously known manifestations [14,35]. Additionally, some LCS patients are disproportionately affected by their social determinants of health (SDOH), due to socioeconomic and political factors as well as behavior and psychosocial background [36]. Up to now, the pathophysiological background of the persistent cardiophysiological and pulmonary abnormalities remains unclear to a certain degree with significant dissociation between symptoms and objective parameters [14]. As a vacillating symptom complex, LCS includes a variable range of associated disorders of multiple organ systems [37], as displayed in Figure 1.

The worldwide prevalence of LCS showed a large variation, ranging from 1% in a Danish study [38] up to 35–77% in a German study [39,40]. Analyzing the circumstances of these variable prevalences, several factors that contribute to the observed variability have to be taken into consideration [14]: the complex interaction within persistent symptoms, severity of acute illness, and the burden of comorbidities might be influenced by several determinants, such as age, sex, timing of assessment, sociodemographic factors, pre-existing health diseases, vaccination status, sample size, pro- or retrospective data assessment, as well as source of enrollment, and type of statistical survey [14,41,42,43].

### 3.2. Estimated Underlying Conditions for Cardiovascular Disorders and LCS

#### 3.2.1. Acute Phase of COVID-19 and Oxidative Stress

Previous research on post-COVID-19 manifestations have elucidated associations between severe clinical illness in the acute phase and severe persistent long-term symptoms in LCS patients [44]. Due to the variability of associated cardiac disorders and symptoms in people with acute COVID-19 and LCS, a deeper discussion on the potential pathophysiological mechanisms in these patients is required [14]. Acute COVID-19-associated cardiophysiological disorders may be based on several pathophysiological pathways and cascades, such as dysregulation of the RAAS system [45], direct cytotoxic injury [46], endotheliitis, and thromboinflammation [33], as well as a dysregulated cytokine response by an altered immune response [32]. In this context, the association between COVID-19 as an endothelial disease and the affected organs, which are perfused by the capillary microcirculation, has to be emphasized [3]. The capillary microcirculation system is based on two cell types, i.e., endothelial cells and pericytes, which express the angiotensin-converting enzyme 2 (ACE-2) protein on their cell membrane, whereby acute SARS-CoV-2 infection has been previously reported upon for its vascular endotheliitis and thrombogenicity in small and large vessels [3,47]. In previous research, SARS-CoV-2 particles have been detected by electron microscopy in the endothelium of different affected organs, such as kidney, brain, heart, lung, and skin [48]. Although acute SARS-CoV-2 primarily affects the pulmonary components, a systemic persistent pan-vascular COVID-19-related involvement has been elucidated before [49,50]. In this context, acute and long-term SARS-CoV-2-related direct or indirect impairment of the endothelial barrier and subsequently endotheliitis and associated multiorgan failure have been reported previously [49]. Various mechanisms contribute to developing endothelial dysfunction, such as hyperinflammation, cytokine storming, cell injury, pyroptosis, persistent oxidative stress, glycocalyx disruption, reduced nitric oxide bioavailability, and thrombogenicity [49].

Given these pathophysiological mechanisms, divergent study results were reported with respect to the prevalence of acute SARS-CoV-2-associated “true” myocarditis. Myocarditis, defined as lymphocytic infiltration and myocyte necrosis, was only evident in 14% of the cases, as described by Basso et al., whereby Lindner et al. reported a high prevalence of viral particles in the heart (59%) and their clinical relevance in a high percentage of the cases (41%) [14,51,52]. On the one hand, this may be due to acute ischemic injury or myocarditis, or additionally caused by infection-associated microthrombosis as reported before [14,53]. Elevated cytokine levels, such as interleukins, interferon (IFN)-γ, or tumor necrosis factor (TNF)-α play an important role in the framework of cardiac damage in SARS-CoV-2 infection with respect to the cytokine storm aggravating endothelial dysfunction, hypercoagulation, and neutrophil infiltration [54,55]. Next to these mechanisms, which display the persistence of acute-phase infection processes, specific pathways representing cardiovascular damage in LCS must be given significant attention: firstly, the persistence of viral reservoirs or secondly, molecular mimicry in the context of an autoimmune response [49].

#### 3.2.2. LCS-Related Cardiovascular and Cardiorespiratory Sequelae

According to the previously described acute effects of SARS-CoV-2 infection on the cardiovascular system, several long-term cardiovascular effects in LCS have been reported [14]. In previously hospitalized patients with COVID-19, excessive risk of major cardiovascular events and an increased risk of developing T2D after COVID-19 infection was reported [14,56]. Furthermore, an increased rate of persistent myocardial inflammation (up to 60%) based on cardiac magnetic resonance imaging (CMR) was seen in this group of patients [57]. Physical inactivity, cytokine storm, unphysiological nutrition, and drugs (e.g., dexamethasone) are just a few reasons underpinning cardiorespiratory limitation post-acute COVID-19, leading to reduction in peak oxygen consumption in these LCS patients [58,59]. Current research revealed persistent pulmonary impairment in previously hospitalized COVID-19 subjects due to endothelial function impairment three months after the acute phase of COVID-19 [50]. Additionally, numerous reports on LCS revealed imbalances in the autonomic nervous system associated with persistent COVID-19 infection, such as orthostatic hypotension, neurocardiogenic syncope, or postural orthostatic tachycardia syndrome (POTS) [60,61,62].

Current scientific research for the specific treatment of LCS and cardiovascular disorders is increasingly being pushed [14]. Individual LCS-associated cardiovascular disturbances include various potential mechanisms such as genetic predispositions, endotheliopathy, obesity, and the role of cytokine storming based on altered immune status [14]. Nevertheless, LCS-associated cardiovascular disorders represent a high symptom burden for many patients, whereby recent studies revealed a certain dissociation between subjective symptoms and objective clinical routine diagnostic measurements [14,58,63]. Therefore, several predictors for non-recovery after hospital admission with COVID-19 within the first 6 months were identified: two or more cardiovascular or metabolic comorbidities, middle age, female sex, or initially severe clinical course of COVID infection [63]. Furthermore, subjects suffering from diabetes were estimated to be at an increased risk for LCS, particularly due to pancreatic *ß*-cell dysfunction and aggravated insulin resistance by peripheral tissue inflammation [64,65].

### 3.3. The Potential Impact of SGLT2 Inhibitors on LCS

In the clinical course and long-term outcomes of LCS, the severity of physical and mental health impairment were closely related [63], so that an interdisciplinary multimodal approach for an effective therapy is urgently needed. The clinical situation of affected LCS patients creates an urgent demand for innovative drugs to treat their various complaints. While drug development is known to be a very slow process, starting with identifying promising candidates in preclinical animal models and a subsequent process to establish an approved drug, elapsing generally more than ten years, it is an established strategy to evaluate the effectiveness of existing approved drugs in the context of long COVID [66]. Up to date, the potential impact of glucose-lowering agents, especially the SGLT2 inhibitors, to potentially modify the symptoms of LCS remain unclear, whereby the current scientific research so far revealed controversial results in acute COVID-19 [67].

#### 3.3.1. Anti-Inflammatory and Beneficial Systemic Effects

**Figure 2:**
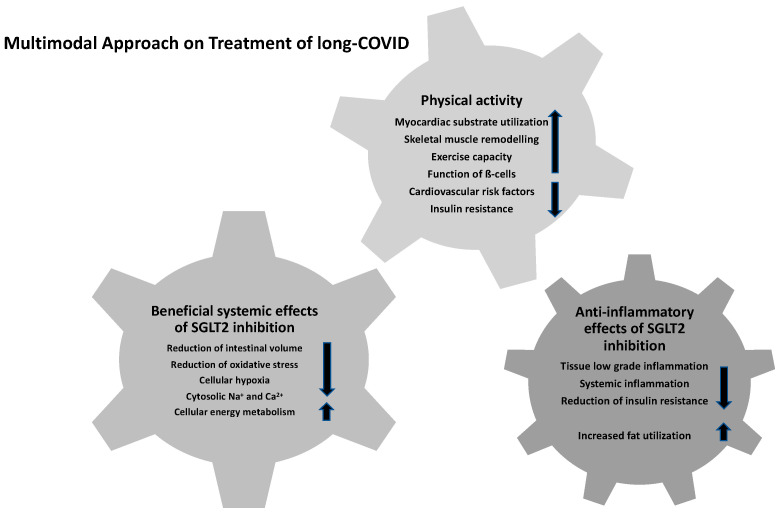
Multimodal approach for the treatment of LCS.

SGLT2 inhibitors may have anti-inflammatory effects by various underlying mechanisms (Figure 2) potentially impacting the different multimodal components of LCS management. Therefore, low-grade tissue inflammation as well as the systemic inflammatory response were shown to be diminished [67]. In this context, chronically increased inflammatory levels as a predisposing fundamental of LCS might be altered positively by the SGLT2 inhibitor empagliflozin, as reported previously [67,68]. Specifically, previous research revealed the positive effects of SGLT2 inhibitors due to reduced inflammation indicators, such as ferritin, interleukin-6 level, and C-reactive protein, and their accompanying reduced risk of SARS-CoV-2-related prothrombogenic issues due to positive effects on the vascular endothelium inflammatory processes [69]. In contrast, the recently published data of the Austrian EMMY trial revealed favorable results with SGLT2 inhibitors for the structural and functional cardiac remodeling in people suffering from an acute myocardial infarction without a reduction in systemic inflammation [70].

Increased fat utilization and the associated reduction in obesity-induced inflammation as well as insulin resistance by M2 macrophage activation are reported to have beneficial effects for the clinical outcomes of persistent COVID-19 [67,71]. While adipose tissue and ectopic fat depots have been considered as a contributor for chronic exaggerated immune activation, viral spread and cytokine amplification are known to be associated with poor prognosis in patients with severe COVID-19 [72,73]. In addition to the proposed impact of SGLT2 inhibitors on the chronic inflammatory cascades, several other effects might beneficially influence the clinical course of LCS: selective reduction of interstitial volume with almost stable blood volume, reduction of oxidative stress and sympathetic activity, enhanced cellular protection by lowering of cytoplasmic natrium and calcium concentrations, alterations in cellular energy metabolism, and decreased cellular hypoxia [67,72,74,75]. Reduced lactate levels and stable maintenance of the cytosolic pH levels are previously described positive effects of dapagliflozin to prevent the severe chronic course of COVID-19 infection and to reduce viral load and chronic inflammatory conditions [76]. Hence, further research was initiated to evaluate the effects of dapagliflozin on respiratory failure in patients with COVID-19 (DARE-19) as a multi-center trial (ClinicalTrials.gov identifier: NCT04350593). In this context, Rossello et al. reported the safe and effective use of SGLT2 inhibitors in hospitalized patients with cardiometabolic risk factors during the COVID-19 pandemic without any significant efficacy in the acute infection phase in the DARE-19 trial [77]. However, the drug was well-tolerated and the observed data revealed no concerns about volume depletion, increased risk of diabetic ketoacidosis (DKA), or acute kidney injury [78]. Furthermore, the results of Li et al. demonstrated that a decrease in ACE expression and accompanying elevated levels of pro-inflammatory chemokines and cytokines in acute COVID-19 patients as well as in SARS-CoV-2-infected cardiomyocytes were estimated to play an important role in acute COVID-19-affected multiple organ dysfunction [79]. Recent data implicated that the subsequent exacerbated inflammatory sequelae, such as endothelial dysfunction, fibrosis, and oxidative stress mediated by SARS-CoV-2 infection, are responsible for the acute COVID-19-associated adverse cardiorenal events [79]. By improving the abnormal apelin-ACE2 signaling, SGLT2 inhibitors might represent a potential therapeutic approach in ameliorating the cardiorenal dysfunction in acute COVID-19 patients and serve as a potential effective LCS treatment option [79]. In addition, SGLT2 inhibition is reported to be associated with optimized myocardial substrate utilization, positive skeletal muscle remodeling, positive effects on vascular function, and improved cardiac function and exercise capacity in HF in the context of LCS [65,80,81]. Hence, these underlying conditions and further meta-analyses suggest the potential beneficial effects of SGLT2 inhibitor treatment to reduce acute and long-term COVID-19 mortality risk in the general population and in those with metabolic diseases [74,82,83]. In addition to this potential new drug option, health care professionals should recommend lifestyle modification such as regular physical exercise and smoking cessation to improve long-term management in people with LCS [79].

#### 3.3.2. Future Innovative Therapeutic Approaches

While LCS represents an emerging chronic illness affecting millions of people worldwide, the scientific community spends a lot of effort in discovering potential effective therapeutical drugs for LCS treatment [66]. In this context, the COVID-OUT trial revealed a significant subsequent risk reduction of 41.3% in LCS for early outpatient COVID-19 treatment with metformin during a 10-month follow-up [66]. Alternative substances, such as fluvoxamine and ivermectin, did not reveal significant benefits in the COVID-OUT trial for LCS risk reduction [66]. Up to date, several initiated trials focusing on the medical treatment options of LCS have been registered on clinicaltrials.gov, mainly focusing on autoantibody neutralization in LCS (BLOC trial, ClinicalTrials.gov identifier: NCT05911009) or magnesium and vitamin D administration in LCS (ClinicalTrials.gov identifier: NCT05630339). In the UK, the multi-center trial HEAL-COVID has been set up to reveal the long-term consequences of COVID-19. The participants were randomized to receive either atorvastatin (40 mg daily for 15 months), the oral anticoagulant apixaban (2.5 mg twice daily for 2 weeks), or medical standard usual care [3]. Atorvastatin has been reported for its favorable pleiotropic effects on the reduction of oxidative stress and vascular inflammation contributing to preserved endothelial function [22]. Additionally, the oral anticoagulant apixaban is being trialed as a prophylactic anticoagulant since the high association between COVID-19 and consecutive thromboembolism and microangiopathy has been proven beforehand [3,84]. Another drug worthy of study in the context of LCS is an angiotensin receptor blocker, such as telmisartan, due to its ability to reestablish cardiovascular homeostasis by regulating the RAAS [3,85]. Therefore, Cooper et al. reviewed the association between LCS and cardiovascular complications and future implications for pharmacological therapies [85].

Up to now, no registered trial is focusing SGLT2 inhibitor administration in LCS and is evaluating the various previously reported anti-inflammatory and cardiorenal beneficial systemic effects of SGLT2 inhibitors and their potential role in LCS risk reduction [28]. Due to the previously described various beneficial effects of SGLT2 inhibition [28], further scientific effort will need to be established in ongoing LCS research.

## 4. Conclusions

Although orally administered SGLT2 inhibitors have been demonstrated to exhibit several favorable effects in the clinical course of T2D and heart failure patients, their rationale has not yet been proven in well-designed randomized controlled trials nor for patients suffering from LCS; however, especially for those living with LCS, this information is crucial and urgently needed. In the absence of long-term data assessment in COVID-19, the various anti-inflammatory and beneficial systemic effects of SGLT2 inhibitors might contribute to playing a promising adjunctive therapy in LCS patients suffering from ongoing systemic inflammation. Nevertheless, the possible risk of DKA associated with severe metabolic disorders in COVID-19 in the acute phases might also be presumed for LCS due to chronic inflammatory conditions. The existing data on the mode of action of SGLT2 inhibitors suggest that it might be worthwhile to investigate this pharmacological class further in people with LCS.

## Data Availability

Not applicable.
